# Radiological appearances of uterine fibroids

**DOI:** 10.4103/0971-3026.54887

**Published:** 2009-08

**Authors:** Wilde Sue, Scott-Barrett Sarah

**Affiliations:** Department of Radiology, Norfolk & Norwich University Hospital, Colney Lane, Norwich, Norfolk, NR4 7UY, United Kingdom

**Keywords:** Uterus, leiomyoma, imaging, sonography, MRI

## Abstract

Uterine fibroids, also known as leiomyomas, are the commonest uterine neoplasms. Although benign, they can be associated with significant morbidity and are the commonest indication for hysterectomy. They are often discovered incidentally when performing imaging for other reasons. Usually first identified with USG, they can be further characterized with MRI. They are usually easily recognizable, but degenerate fibroids can have unusual appearances. In this article, we describe the appearances of typical and atypical uterine fibroids, unusual fibroid variants and fibroid mimics on different imaging modalities. Knowledge of the different appearances of fibroids on imaging is important as it enables prompt diagnosis and thereby guides treatment.

## Introduction

Uterine fibroids, also known as leiomyomas or myomas, are the commonest uterine neoplasms. They are benign tumors of smooth muscle origin, with varying amounts of fibrous connective tissue.[1] Fibroids usually arise in the myometrium but may occasionally be found in the cervix, broad ligament or ovaries.[1,2] They are multiple in up to 84% of women.[3] Fibroids have been reported to occur in up to 70% of women by the age of 50 years[4] and are especially common in black women, who also often have more severe disease.[4,5] These benign tumors are hormone dependent, responding to both estrogen and progesterone[6]; they often increase in size during pregnancy and usually decrease in size after menopause. Early age at menarche and obesity are risk factors for the development of fibroids, likely due to the increased exposure to estrogen.[7]

The majority of women with fibroids are asymptomatic; however, 20–50% of them have symptoms such as menorrhagia, pelvic pain and infertility, or complications during pregnancy.[8–10] A large fibroid can present as an abdominal mass or with symptoms secondary to mass effect, e.g., constipation and urinary frequency or retention. Rarely, the patient may present with hydronephrosis or bowel obstruction.[11]

The presence of symptomatic fibroids is the commonest indication for hysterectomy, accounting for approximately one-third of those performed.[12] Traditionally, treatment has been surgical but, in recent years, treatment with uterine artery embolization (UAE) has been increasingly performed and has been shown to be an effective alternative to traditional surgery.[12,13] Other treatment options include myomectomy or administration of gonadotrophin-releasing hormone (GnRH) analogs.[12,14] A relatively new non-invasive technique for treating fibroids is MRI-guided focused ultrasound ablation, which targets high energy ultrasound waves onto a fibroid, leading to localized heating and subsequent cell death.

MRI is the preferred imaging modality for characterizing uterine fibroids and identifying their exact anatomical location, though initial identification is usually by USG. Often, fibroids may also be found incidentally on plain radiographs or CT scans done for other indications.

## Classification and histopathological features

**Figure 1:**
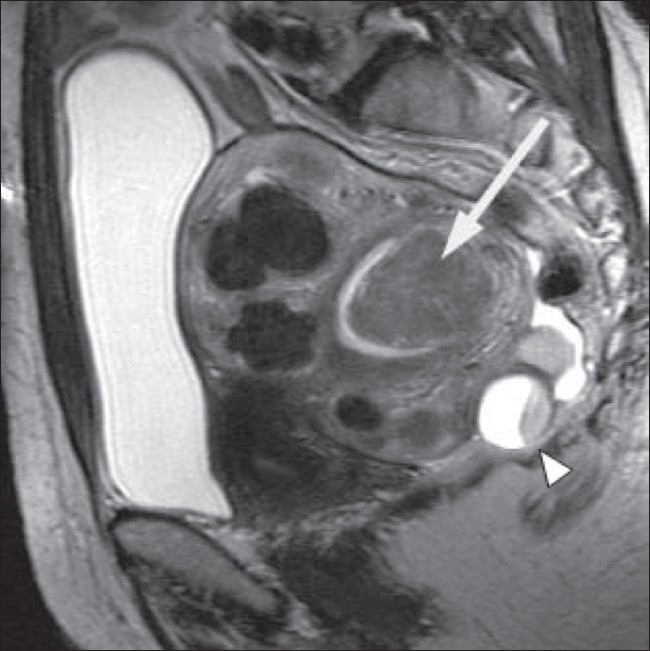
A 51-year-old woman with a history of menorrhagia. Sagittal T2W MRI shows a bulky retroverted uterus containing multiple intramural fibroids and a large submucosal fibroid (arrow) projecting into the endometrial cavity. A complex ovarian cyst is also incidentally demonstrated posterior to the uterus (arrowhead)

**Figure 2:**
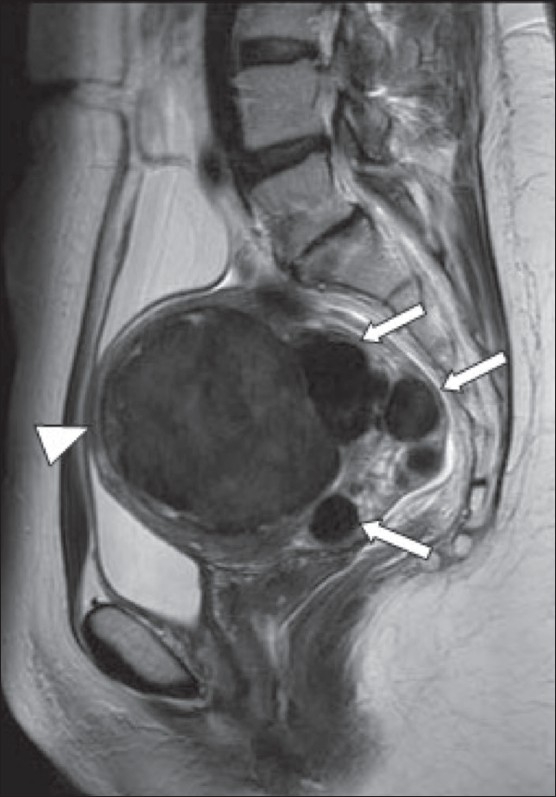
A 43-year-old woman with menorrhagia. Sagittal T2W MRI image shows multiple intramural fibroids (arrows); the largest (arrowhead) lying anteriorly measures 8.5 cm. These show typical low-signal intensity

**Figure 3:**
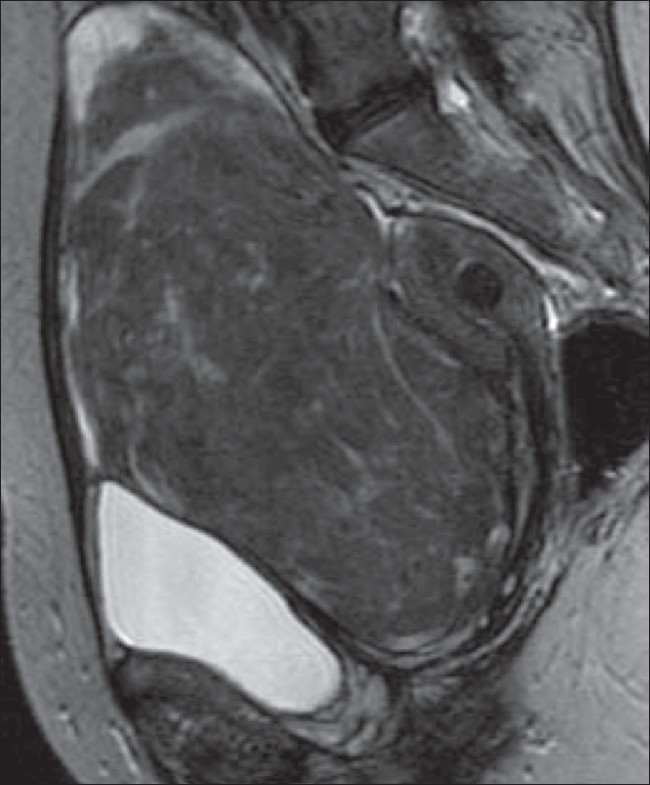
A 59-year-old woman with an abdominal mass and discomfort. Sagittal T2W MRI image shows a 15-cm pedunculated, subserosal fibroid arising from the anterior uterus. There is also a small intramural fibroid lying posteriorly

Uterine fibroids are classified according to their location as submucosal, intramural or subserosal.[15] Submucosal fibroids [Figure 1] are the least common type, accounting for just 5% of all fibroids,[8] but they are the most likely to be symptomatic since they project into the endometrial cavity. Submucosal fibroids can occasionally become pedunculated and prolapse into the cervical canal or vagina.[16] Intramural fibroids [Figure 2] are the most common type, but they are usually asymptomatic; however, they may cause infertility due to compression of the fallopian tubes. Subserosal fibroids, project exophytically into the abdomen or pelvis and can also become pedunculated [Figure 3], which may be confused with ovarian tumors. Pedunculated subserosal fibroids can undergo torsion and consequent infarction and thus be a cause of severe abdominal pain.[8,17] Large fibroids often degenerate as they outgrow their blood supply. The various types of degeneration include hyaline, myxoid, cystic and red degeneration.[8,18,19] Calcification tends to occur following necrosis.[19]

Although the majority of fibroids are benign, it is thought that some uterine leiomyosarcomas arise in a subset of fibroids.[7] Only about 0.23–0.7% of apparently benign uterine fibroids turn out to be leiomyosarcomas on pathological examination.[20,21] Most leiomyosarcomas arise *de novo*. A leiomyosarcoma can be difficult to distinguish from a benign fibroid and this possibility should always be considered in a patient with a rapidly growing uterine fibroid.

## Imaging characteristics

### Plain radiography

**Figure 4 (A, B):**
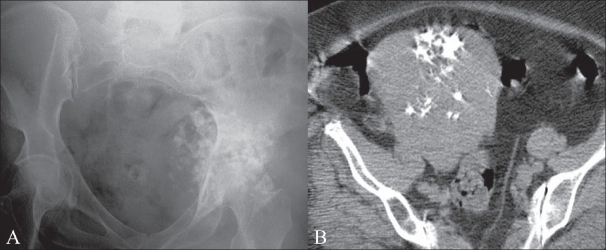
A 58-year-old woman with sacral pain. A frontal pelvic radiograph (A) shows calcification overlying the left hip, initially thought to be suggestive of a chondrosarcoma. A subsequent CT scan (B) reveals an incidental calcified fibroid

Fibroids are usually only identified on plain radiographs if they are calcified and sometimes large fibroids may be seen as soft tissue or calcified masses displacing bowel gas. Occasionally, a calcified fibroid may be mistaken for more sinister pathology [Figure 4].

### USG

USG is usually the initial investigation for examining the female pelvis. Ideally, both transabdominal (TA) and transvaginal (TV) scans should be performed. Transvaginal scans are more sensitive for the diagnosis of small fibroids; however, when the uterus is bulky or retroverted, the uterine fundus may lie outside of the field of view. Transabdominal views are often of limited value if the patient is obese. Ultrasonography is highly operator dependent and in skilled hands, fibroids as small as 5 mm can be demonstrated on TV USG.

**Figure 5 (A, B):**
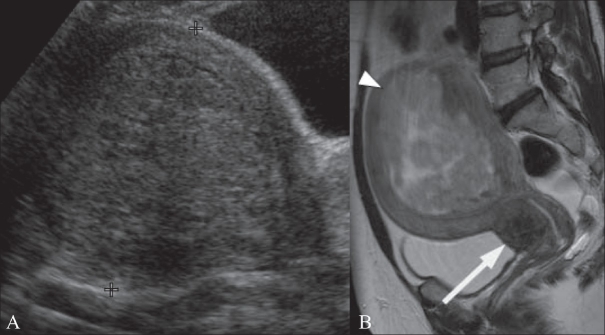
A 49-year-old woman with a history of menorrhagia. Transabdominal (TA) USG image (A) shows a bulky uterus showing a 10-cm submucosal fibroid (between cursors). Sagittal T2W MRI image (B) in the same patient shows that the submucosal fibroid (arrowhead) is heterogeneous indicating degeneration. There is also a 2.5-cm cervical fibroid (arrow)

**Figure 6:**
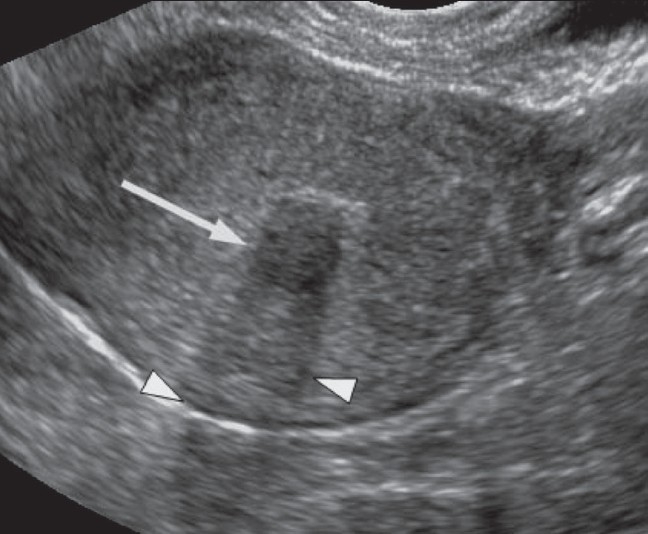
A 46-year-old woman with a history of abdominal pain. Transvaginal (TV) USG image shows a 1.1-cm submucous fibroid (arrow) with posterior acoustic shadowing (arrowheads)

**Figure 7 (A, B):**
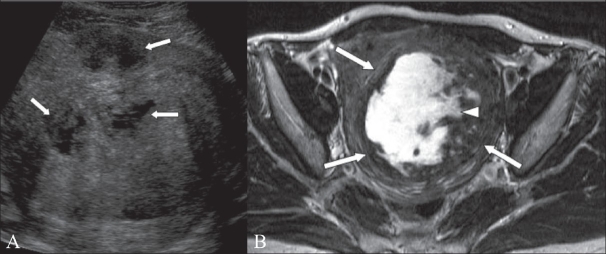
A 46-year-old woman with menorrhagia. TA USG image (A) shows a 7-cm intramural fibroid containing cystic areas (arrows). Axial T2W MRI image (B) shows an 11 × 8 cm fibroid (arrows) containing central high signal, consistent with cystic degeneration (arrowhead)

Typically, fibroids appear as well-defined, solid masses with a whorled appearance. These are usually of similar echogenicity to the myometrium, but sometimes may be hypoechoic. They cause the uterus to appear bulky [Figure 5] or may cause an alteration of the normal uterine contour. Even noncalcified fibroids often show a degree of posterior acoustic shadowing [Figure 6], though this is of course more marked in calcified fibroids. Degenerate fibroids may have a complex appearance, with areas of cystic change [Figure 7]. Doppler USG typically shows circumferential vascularity; however, fibroids which are necrotic or have undergone torsion will show absence of flow.[17]

Submucous fibroids are usually clearly visible separate from the endometrium at TV USG, but can be difficult to differentiate from polyps. Sonohysterography is a technique in which sterile saline is instilled into the uterine cavity via a transcervical catheter whilst performing a TV scan. This method allows better visualization of the endometrium and has been shown to be more accurate than traditional TV USG in detecting submucous fibroids and in differentiating them from polyps.[22]

Large fibroids can occasionally cause obstruction of the ureters, with secondary hydronephrosis. Therefore, USG examination should include the urinary tract whenever a large pelvic mass is identified. The diagnosis of fibroids on USG is usually reasonably straightforward though focal adenomyosis can mimic a fibroid and a pedunculated uterine fibroid can sometimes be mistaken for an adnexal mass.[23] When there is doubt about the origin of a pelvic mass at USG, further evaluation with MRI should be performed.

### CT Scan

**Figure 8:**
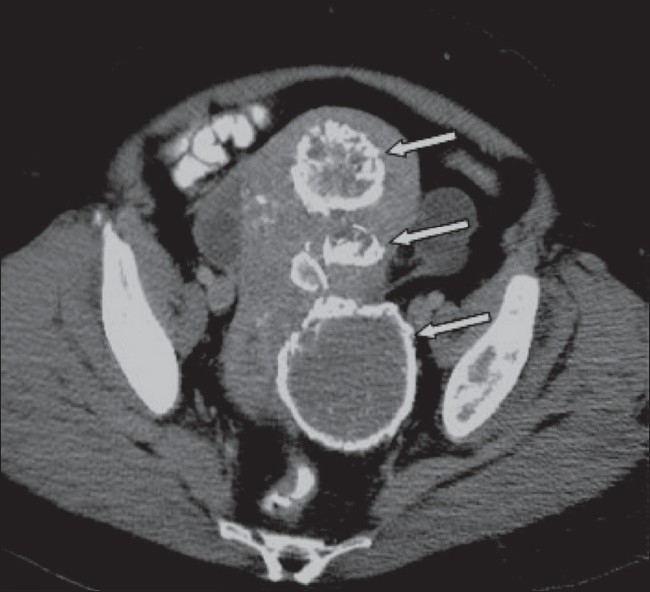
A 51-year-old woman known to have fibroids, which have been treated by uterine artery embolization 18 months earlier. Axial CT scan shows several fibroids with peripheral calcification (arrows)

CT scan is not the investigation of choice for the characterization of pelvic masses. Uterine fibroids are often seen incidentally on CT scans performed for other reasons. The typical finding is a bulky, irregular uterus or a mass in continuity with the uterus. Degenerate fibroids may appear complex and contain areas of fluid attenuation. Calcification is seen in approximately 4% of fibroids[18] and is typically dense and amorphous. However, calcification can also be confined to the periphery of the fibroid [Figure 8], when it is thought to be secondary to thrombosed veins from previous red degeneration.[18]

**Figure 9:**
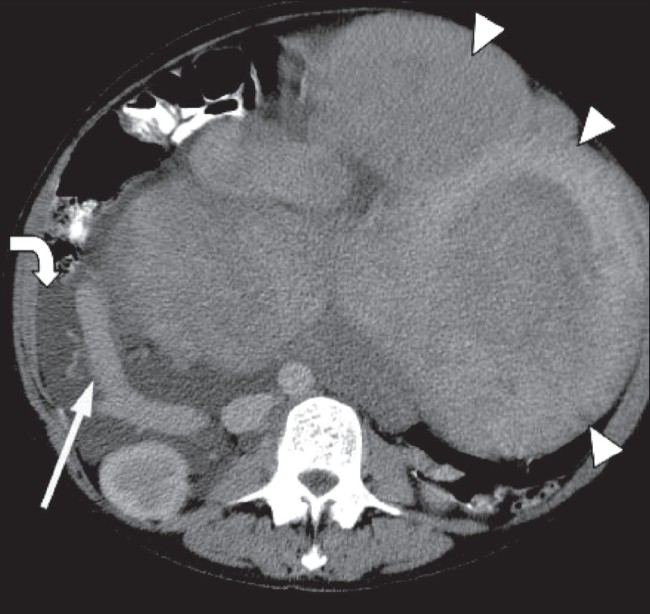
A 45-year-old woman with a large, asymptomatic abdominal mass. Axial CT scan image shows a 30-cm heterogeneous mass (arrowheads) that extends up to the epigastrium. The right ovarian vein is dilated (arrow) and there is mild right hydronephrosis. There is also free intra-abdominal fluid (curved arrow). Histology confirmed this to be a massive, partly degenerate fibroid

On contrast-enhanced scans, fibroids usually show low attenuation relative to the myometrium although, occasionally, they may be of the same or of higher attenuation.[24] If the fibroid has undergone acute torsion, there may be enhancement of the rim of the fibroid due to obstructed peripheral veins but there will be no enhancement centrally.[17] Fibroids can occasionally grow to massive sizes [Figure 9] and present with symptoms secondary to mass effect, such as hydronephrosis. Fibroids are a rare cause of pseudo-Meig's syndrome and can occasionally present with ascites.[25]

### MRI

**Figure 10 (A, B):**
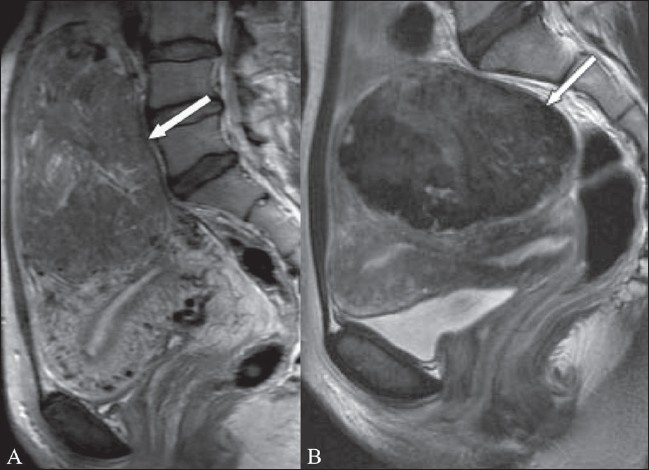
A 37-year-old woman with menorrhagia. Sagittal T2W MRI (A) shows an 18-cm, broad-based, subserosal fibroid (arrow). Repeat sagittal T2W image (B), 3 years post-UAE shows good response to treatment: the fibroid has reduced in size, now measuring 10.5 cm in AP dimension (arrow)

MRI is the preferred method for accurately characterizing pelvic masses. It has been shown to be more sensitive in identifying uterine fibroids than USG,[26–28] it does not involve the use of ionizing radiation, and it can readily demonstrate the uterine zonal anatomy. Submucosal, intramural and subserosal fibroids are usually easily differentiated with MRI, and fibroids as small as 5 mm in diameter can be demonstrated. Fibroids in relatively unusual locations, such as within the cervix, can also be identified [Figure 5]. MRI is also used to both predict and to assess the response of fibroids to UAE [Figure 10].

On T2W images, the normal endometrium is of high signal intensity. Surrounding the endometrium, is a low-signal band known as the junctional zone, which represents the inner myometrium. The remainder of the myometrium is of intermediate signal on T2W images. [15]

MRI sequences should include axial and sagittal T2W images as well as T1W images in at least one plane. The routine use of gadolinium has been shown not to contribute to either fibroid detection or characterization.[29] However, gadolinium can be used to determine vascularity when assessing the suitability of a fibroid for UAE.

**Figure 11:**
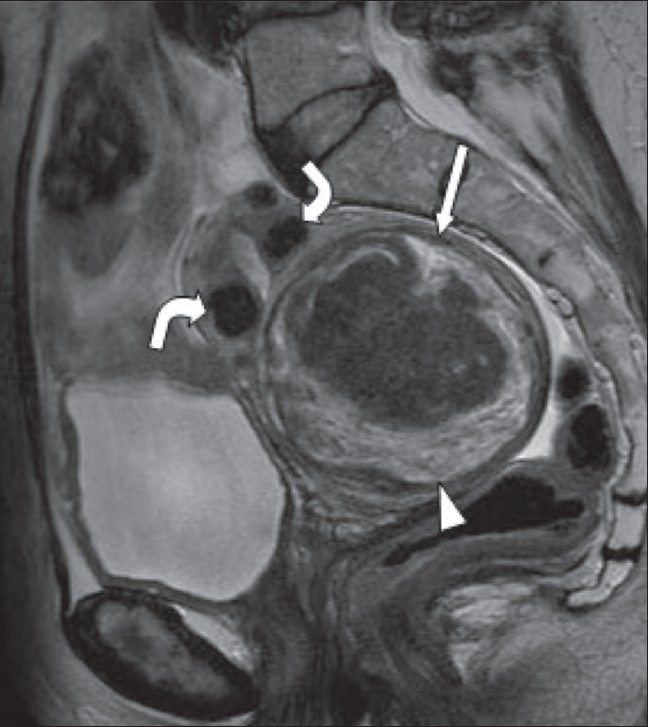
A 71-year-old woman shown to have uterine fibroids whilst undergoing an MRI scan of her hip. She had no gynecological symptoms. Sagittal T2W MRI image shows a 7-cm intramural fibroid (arrow), with surrounding high signal (arrowhead). Several other small intramural fibroids (curved arrows) and a trace of fluid in the pouch of Douglas are also present

Typically, non-degenerate fibroids are well-defined masses of low-signal intensity as compared to the myometrium on T2W images [Figure 2] and isointense to the myometrium on T1W images.[8,18] Uterine fibroids may have a high-intensity rim on T2W images [Figure 11], which has been shown to be secondary to a zone of dilated lymphatic vessels, dilated veins, edema or a combination of these.[30] Fibroids may show degeneration due to inadequate blood supply and then have a variable and often heterogeneous appearance, with minimal or irregular enhancement. The commonest type of degeneration is hyaline; it accounts for approximately 60%[19] of all degeneration and produces low signal on T2W images,[18,19] with no enhancement. Fibroids with hyaline or calcific degeneration are difficult to distinguish from non-degenerated fibroids on MRI. Areas of calcification can appear as signal voids on MRI.

**Figure 12 (A, B):**
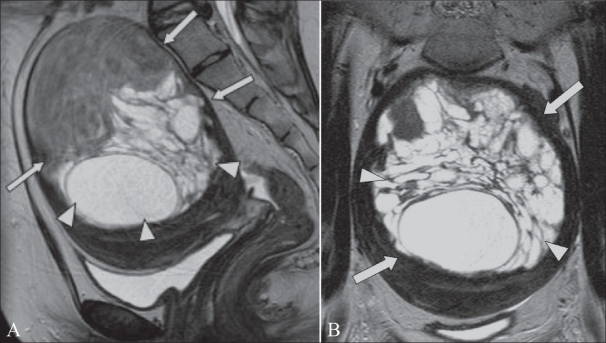
A 21-year-old woman with menorrhagia and a pelvic mass. Sagittal (A) and coronal (B) T2W MRI images show a large intramural fibroid (arrows) that contains septate areas of high signal (arrowheads). The patient underwent a myomectomy and histopathology confirmed that this was a fibroid with myxoid degeneration

Cystic degeneration [Figure 7] occurs in approximately 4% of fibroids[18] and typically occurs after hyaline degeneration.[19] It usually results in high-signal intensity on T2W images and low-signal on T1W images.[8,18] Fibroids that have undergone myxoid degeneration are filled with a gelatinous material and can be difficult to differentiate from fibroids that have undergone cystic degeneration; however, they typically appear as more complex cystic masses [Figure 12]. The appearance of myxoid degeneration is important to recognize as it may also be seen in leiomyosarcomas.[18]

**Figure 13 (A, B):**
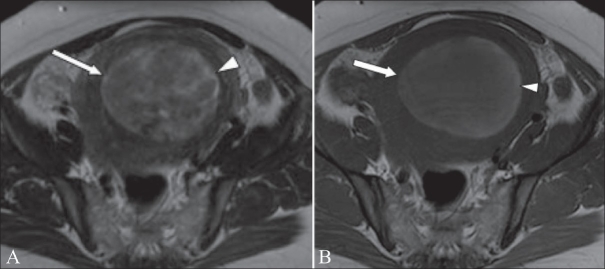
A 44-year-old woman, known to have uterine fibroids, admitted with acute abdominal pain and vaginal bleeding. Axial T2W (A) and T1W (B) MRI images show a heterogeneous mass (arrow) within the endometrial cavity. The mass shows peripheral high signal (arrowheads) on the T1W image, due to hemorrhage. The patient underwent a hysterectomy, and histology showed that this was a submucosal fibroid that had undergone extensive red degeneration

Red, or carneous degeneration [Figure 13], is secondary to hemorrhagic infarction due to obstructed draining veins. This usually occurs in pregnancy or with the use of oral contraceptives and patients can present with an acute abdomen. The fibroid may have a peripheral rim of low signal on T2W images and high signal on T1W images due to the peripheral obstructed veins.[18] Areas of hemorrhage within the fibroid produce varying signal, depending on the age of the blood products. Recent hemorrhage shows high signal on both T1W and T2W images; there will be no enhancement as the blood supply is obstructed.

Fibroids that demonstrate high signal on T1W images prior to embolization are likely to have a poor response to UAE as they may already have outgrown their blood supply and undergone hemorrhagic necrosis. High signal on T2W images prior to embolization has been shown to be a predictor of good response.[31] The vascularity of a fibroid is demonstrated by gadolinium enhancement and this is also a predictor of good response to UAE. Post-UAE fibroids typically show high signal on T1W images due to hemorrhagic necrosis.

### Hysterosalpingography

Hysterosalpingography (HSG) is usually performed to assess tubal patency in patients with infertility. However, it is also useful for evaluating the contour of the endometrial cavity, and submucous fibroids can be demonstrated as filling defects. Occasionally a submucosal fibroid may cause tubal obstruction. Intramural or subserosal fibroids will not be seen with this technique.

## Unusual fibroid variants

### Diffuse leiomyomatosis

**Figure 14:**
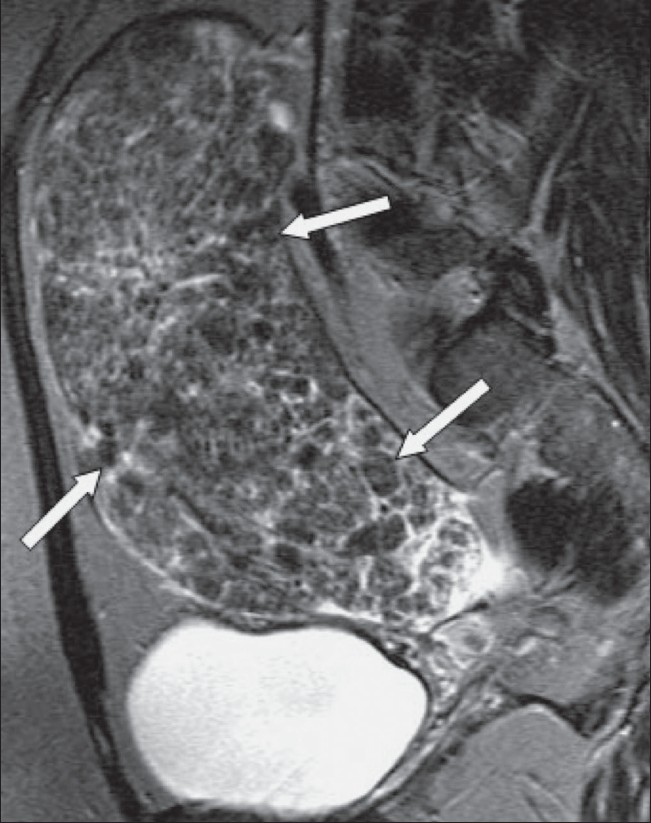
A 47-year-old woman with a pelvic mass. Sagittal T2W MRI image shows an enlarged heterogeneous uterus containing multiple nodules (arrows). Hysterectomy and histology showed that this was diffuse leiomyomatosis. There was no evidence of extra-uterine spread

Diffuse leiomyomatosis [Figure 14] is a rare condition that consists of diffuse involvement of the myometrium by innumerable small fibroids, which results in symmetrical enlargement of the uterus. Although histologically benign, there may be dissemination through the peritoneal cavity or occasionally metastases to distant organs.[18]

### Lipoleiomyomas

**Figure 15 (A-D):**
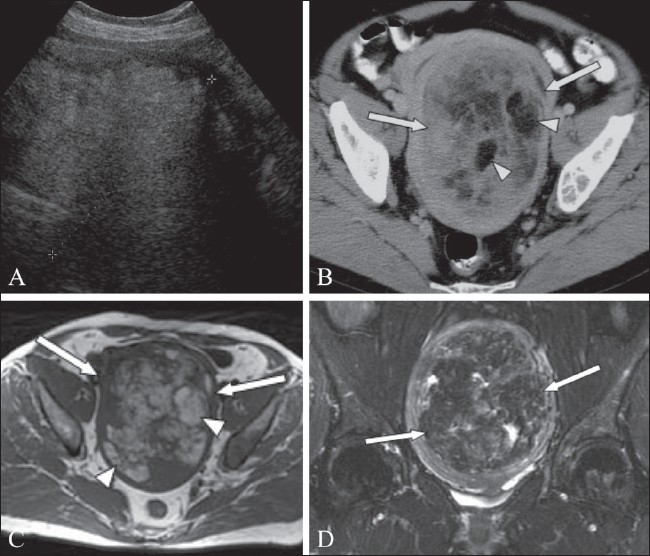
A 65-year-old woman with a pelvic mass. Transabdominal USG image demonstrates an echogenic mass (between cursors) within the pelvis. Axial CT scan (B) shows a 10 × 15 cm mass (arrows) containing areas of fat attenuation (arrowheads). Axial T1W MRI image (C) shows that the mass (arrows) is heterogeneous, containing areas of high signal (arrowheads). Coronal fat-saturated T2W MRI image (D) confirms the presence of fat (arrows). Histological examination identified this to be a lipoleiomyoma

Lipoleiomyomas [Figure 15] are rare fat-containing fibroids, with a reported prevalence of between 0.005 and 0.2%.[32] They are benign and present with the same symptoms as uterine fibroids. The most likely cause is thought to be fatty metamorphosis of the smooth muscle cells of a leiomyoma.[33] If pedunculated, they can be mistaken on imaging for ovarian dermoids. At USG, they are typically echogenic masses, in contrast to the usually hypoechoic fibroids. On MRI, these tumors typically show high signal on both T1W and T2W images; often, they have a hypoechoic rim, which is thought to be due to a surrounding layer of myometrium.[33] A fat-suppression technique is useful to confirm the presence of fat.

## Differential diagnosis

### Leiomyosarcomas

**Figure 16 (A,B):**
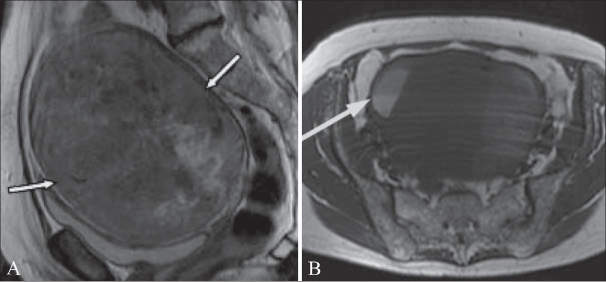
A 70-year-old woman with postmenopausal bleeding and a pelvic mass. Sagittal T2W MRI image (A) shows a 13.5-cm heterogeneous pelvic mass (arrows). Axial T1 MRI image (B) shows areas of high signal within the mass, including an area with a fluid-fluid level (arrow) that is in keeping with hemorrhage. The patient underwent a hysterectomy and this was confirmed to be a leiomyosarcoma

Leiomyosarcomas [Figure 16] may occasionally arise in a preexisting fibroid but are usually known to occur *de novo*.[7] The patient classically presents with a pelvic mass that has shown a recent or rapid increase in size.[20] They are typically large, heterogeneous masses containing areas of hemorrhage.[15] They usually undergo marked post-contrast enhancement and have more ill-defined, irregular margins than benign uterine fibroids.[15,34]

### Adenomyosis

**Figure 17:**
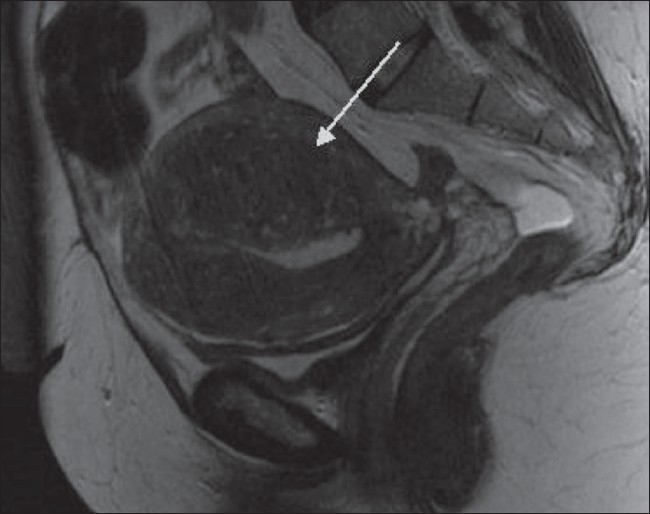
Sagittal T2W MRI image shows thickening of the junctional zone in a patient with adenomyosis. The thickening is most marked posteriorly (arrow) and several small cystic spaces can be seen within it

Adenomyosis is the presence of heterotopic endometrium within the myometrium and is associated with adjacent myometrial hyperplasia. Adenomyosis is more common in multiparous women. It typically presents with symptoms similar to those caused by uterine fibroids, and the two conditions can also coexist.[29] Adenomyosis is usually a diffuse condition, which is seen on MRI as a thickened junctional zone [Figure 17]. It is usually easy to differentiate from uterine fibroids as adenomyosis typically has multiple small hyperintense foci on T2W imaging, which represent glandular structures. However, occasionally adenomyosis presents as a more focal mass and can then be mistaken for a fibroid. Typically, focal adenomyosis is an ill-defined, oval-shaped, low-signal mass on T2W MRI, whereas fibroids are usually well defined and round in shape.[8] Uterine fibroids are also often accompanied by dilated veins, whereas focal adenomyosis is not.[35]

### Endometrial Polyps

Endometrial polyps can present with post-menopausal bleeding, however they are often an incidental finding on TV USG. They typically appear as pedunculated endometrial masses and will usually demonstrate an intact overlying endometrial stripe. Differentiation from a submucosal fibroid can however be difficult and sonohysterography can aid diagnosis.

### Ovarian tumors

A very large fibroid or a pedunculated subserosal fibroid can sometimes be mistaken for an ovarian tumor. Ovarian Brenner tumors and fibrothecomas are benign tumors that also show low signal on T2W imaging due to their large fibrous component.[8] Occasionally, the correct diagnosis is not made until surgery.

### Transient myometrial contractions

Myometrial contractions typically appear as low-signal focal masses on T2W images and can be mistaken for uterine fibroids. However, as the contractions only occur transiently, the masses are not consistent and will usually have disappeared on subsequent sequences.[36] If still in doubt, the T2W sequence can be repeated.

## Conclusion

Uterine fibroids are common tumors and although benign they can be associated with significant morbidity. They may be encountered incidentally when performing imaging for other reasons and are usually easily recognizable. However, degenerate fibroids can have unusual appearances. Awareness of the various appearances enables a prompt diagnosis and can guide treatment.
